# Monolithic Axial InGaAs Quantum Dot Emitters in GaAs-Based Nanowires via Sb-Mediated Facet Engineering

**DOI:** 10.1021/acs.nanolett.6c02123

**Published:** 2026-07-02

**Authors:** Hyowon W. Jeong, Aris Koulas-Simos, Imad Limame, Markus Döblinger, Sang Kyu Kim, Chirag C. Palekar, Jonathan J. Finley, Stephan Reitzenstein, Gregor Koblmüller

**Affiliations:** † Walter Schottky Institute, TUM School of Natural Sciences, 9184 Technical University of Munich, Garching 85748, Germany; ‡ Institute for Physics and Astronomy, 26524 Technical University Berlin, Berlin 10623, Germany; § Department of Chemistry, 9183 Ludwig-Maximilians-University of Munich, Munich 81377, Germany; ∥ Walter Schottky Institute, TUM School of Computation, Information and Technology, Technical University of Munich, Garching 85748, Germany; ⊥ Department of Physics, 1438 University of California, Berkeley, Berkeley, California 94720, United States

**Keywords:** nanowire heterostructure, InGaAs, Sb surfactant, transmission electron microscopy, twin defects, photoluminescence, cathodoluminescence, single-photon emission

## Abstract

GaAs-based nanowires hosting active quantum heterostructures provide a promising route toward monolithic integration of single-photon sources on silicon, a key requirement for scalable quantum photonics. However, ultrathin axial quantum-emitter formation is often hindered by facet-dependent growth dynamics and rotational twins, which induce lateral overgrowth and compromise interface abruptness. Here, we develop InGaAs-based quantum emitters by tailoring facet evolution via dilute Sb incorporation, which efficiently suppresses twins and promotes confined axial insertion at the growth-front facet. This approach significantly enhances the probability of obtaining abrupt, few-nanometer-thin quantum dots at the nanowire tip. Single-nanowire optical spectroscopy reveals intense, spatially localized emission from the active region with lifetimes as short as (0.51 ± 0.02) ns, and second-order photon-correlation measurements consistently exhibit pronounced antibunching with *g* ^(2)^(0) < 0.4, confirming single-photon emission. These results establish a strong correlation between twin density and axial heterostructure formation, identifying defect control as a key factor in realizing monolithically integrated nanowire single-photon sources.

## Graphical abstract

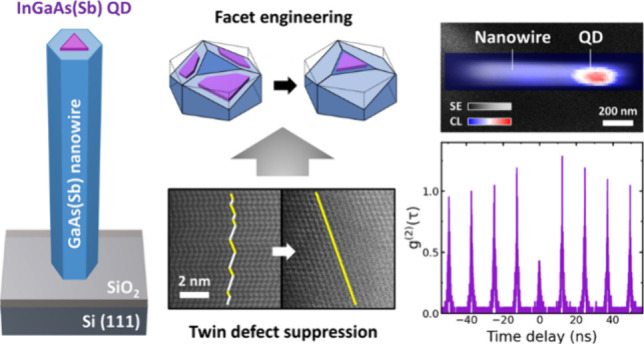

The realization of scalable quantum photonic technologies requires high-purity, deterministic single-photon sources compatible with silicon (Si)-based platforms.^1^ While site-controlled quantum dots (QDs) have demonstrated excellent scalability, uniformity, and device integration,^2–4^ monolithic growth on Si remains challenging due to the large lattice and polarity mismatch, e.g., between GaAs and Si, requiring polar buffer and strain-reducing layers to suppress defect formation and enable high-quality epitaxy.^5^ In this context, free-standing III–V semiconductor nanowires (NWs) have emerged as a powerful bottom-up platform for realizing a variety of nanoscale electronic, optoelectronic, and integrated quantum photonic devices.^6–13^ Their compatibility with lattice-mismatched substrates such as Si^14,15^ as well as the efficient optical mode confinement^16,17^ and the ability to host axial heterostructures in NWs with strain relaxation conditions well beyond the critical thickness limit of planar structures^18^ collectively underpin their versatility.

In particular, quantum heterostructures embedded along the NW axis offer unique opportunities for site-controlled and scalable single-photon sources, combining strong carrier confinement with efficient photon extraction along the NW waveguide.^17,19,20^ Furthermore, NW-based QDs grown along the [111] direction can exhibit highly symmetric confinement potential, which suppresses the fine-structure splitting (FSS) arising from structural or compositional asymmetries.^21^ The near-elimination of FSS makes such NW-QDs promising platforms for the generation of polarization-entangled photon pairs.^22,23^

Recent progress in III–V heterostructure NW-QD platforms has revealed highly promising quantum emission performance, particularly in devices realized through droplet-assisted vapor–liquid–solid (VLS) growth. InP/InAsP NW-QDs, for instance, have achieved nearly ideal single-photon purity with *g* ^(2)^(0) < 0.05 ^24–26^ and improved photon indistinguishability^25,27^ as well as high brightness^25^ and a high degree of entanglement.^23^ AlGaAs/GaAs NW-QDs have likewise shown decent single-photon purity with *g* ^(2)^(0) = 0.19 together with high brightness levels.^28^ However, conventional VLS growth, which relies on a liquid droplet (e.g., Au or self-induced group-III elements), often encounters droplet-related limitations in morphology and structural uniformity and abrupt heterointerface formation,^29–32^ while foreign metal catalysts can additionally introduce contamination into the device structure.^33^

Catalyst-free vapor–solid (VS) growth via selective-area epitaxy (SAE) offers several key advantages for quantum emitters in monolithic NW devices, given the droplet-free growth and compatibility with complementary metal oxide semiconductor (CMOS) technology for integration on Si platforms. This approach has demonstrated well-defined axial quantum heterostructures in various III–V NW material systems, including GaAs/InGaAs, GaAs/AlGaAs, and GaAs/GaAsP.^34–45^ Here, GaAs/InGaAs-based axial NW-QD emitters are particularly attractive due to their excellent optical properties,^34–39^ while the InGaAs material system also offers potential for single-photon sources at telecommunications wavelengths.^1,46^ Achieving the precise thickness control and abrupt interfaces required for well-defined GaAs/InGaAs QDs or quantum disks still remains a key challenge toward deterministic single-photon sources based on such axial inserts.

Bottom-up GaAs-based NWs naturally grow along the epitaxial [111]­B direction with sidewall facets and other minor inclined facets near the growth front that belong to the {1̅1̅0} family of planes. A major obstacle in tailoring the size and shape of axially embedded QD emitters arises from the complex, facet-dependent morphology evolution inherited from the underlying (111)B GaAs-based NW core.^47–51^ In the so-called twin-induced growth mechanism, which also governs InGaAs segment formation,^45^ a strong interplay between facet-dependent growth kinetics and rotational twin formation exists that critically shapes the evolution of the NW growth front.^49,50^ In particular, at high twin densities, subsequent layer deposition tends to enhance lateral infilling over the inclined {1̅1̅0} facets adjacent to the NW growth front.^47,50^ During InGaAs-based segment insertion, this growth mode results in unintended preferential deposition on these inclined facets rather than on the flat (111)­B top facet, thereby reducing growth selectivity.^44^ Consequently, structural and compositional nonuniformities arise in the segment, hindering the realization of ultrathin axial quantum structures with abrupt heterointerfaces and well-defined emission characteristics.

Previous studies have shown that a small molar fraction (≈2–3%) of antimony (Sb) acts as an effective surfactant during catalyst-free GaAs-based NW growth, modifying surface reconstruction, crystal-phase stability, and adatom diffusion dynamics, which in turn significantly improve the structural and optical properties.^52–54^ Notably, precise tuning of the Sb content was found to be decisive in tailoring the rotational twin density,^53^ which is essential for regulating facet evolution at the growth front and restoring growth selectivity.

Here, we exploit Sb surfactant-induced twin suppression in catalyst-free SAE growth to realize ultrathin InGaAs-based quantum-confined heterostructures and QDs embedded in GaAs-based NWs (hereafter referred to as InGaAs­(Sb) and GaAs­(Sb), respectively). By confining the axial insertion thickness to the few-nm length scale through Sb-mediated facet engineering, we establish a defect-minimized growth window that yields InGaAs­(Sb) segments with abrupt compositional and structural transitions at the heterointerface. Structural analysis by scanning transmission electron microscopy (STEM) combined with energy-dispersive X-ray spectroscopy (EDXS) reveals a strong correlation between axial insertion characteristics and twin defect density. Cathodoluminescence (CL) and microphotoluminescence (μPL) measurements along with Hanbury Brown and Twiss (HBT) experiments on individual NWs are further used to investigate the emission in terms of intensity and spatial localization as well as the single photon emission characteristics from the InGaAs­(Sb) active region at the NW tip. Second-order photon-correlation *g* ^(2)^(τ) measurements exhibit pronounced antibunching, confirming the QD nature of the InGaAs­(Sb) region and its suitability as a single-photon source.

Figure 1a schematically displays the designed NW heterostructure in which an InGaAs­(Sb) QD emitter is axially embedded within a GaAs-based NW grown directly on a SAE-prepatterned SiO_2_/Si­(111) substrate using molecular beam epitaxy (MBE). As detailed in S1. Methods (Supporting Information), a small fraction of Sb (≈3–4%) is incorporated into both the GaAs­(Sb) core and the InGaAs­(Sb) insert, while the shell consists of Al_0.3_Ga_0.7_As/GaAs surface passivation layers.

**Figure 1:**
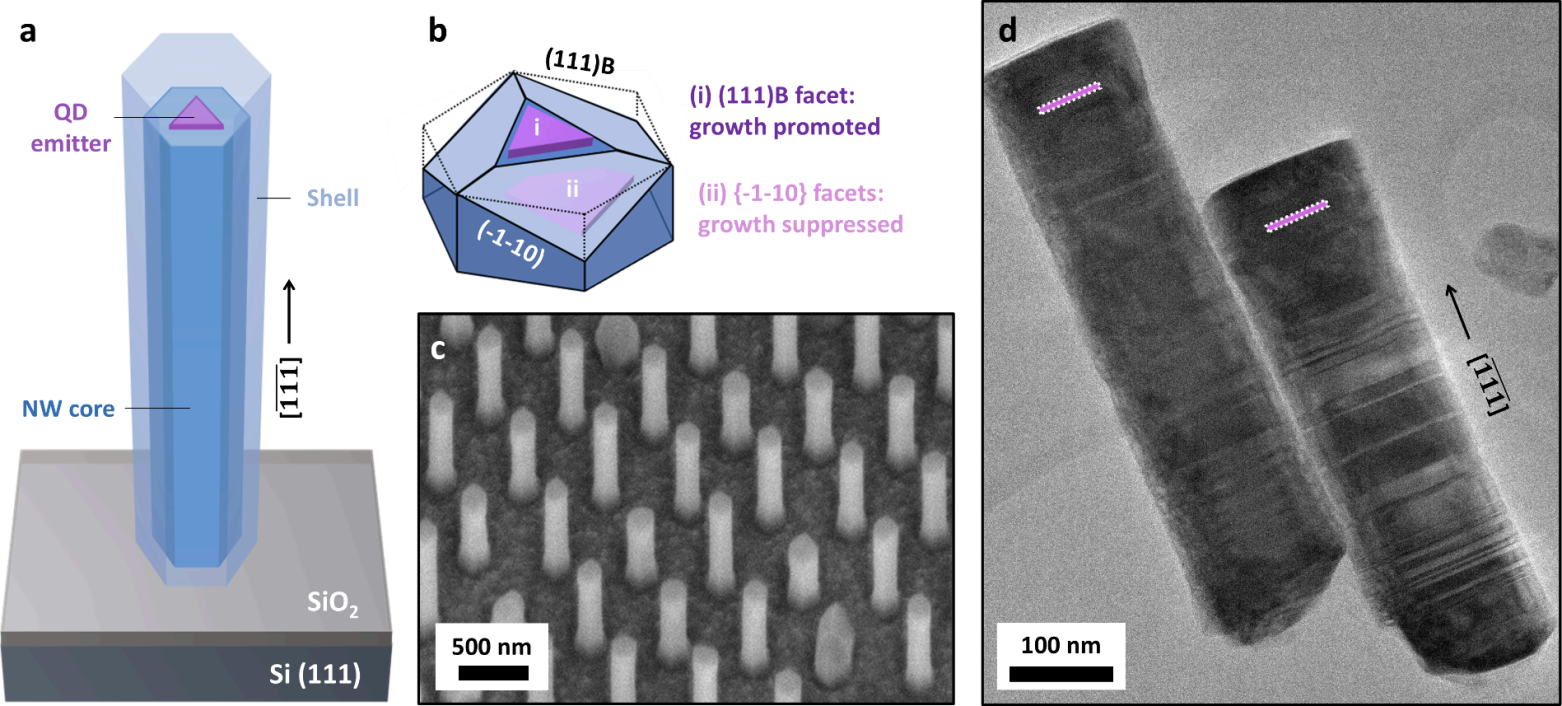
Growth concept and structural overview of axially embedded QDs in NWs. (a) Schematic illustration of an InGaAs-based QD axially embedded within a GaAs-based NW directly grown on a SAE-prepatterned SiO_2_/Si­(111) substrate. (b) Magnified schematic of the InGaAs­(Sb) region, where axial growth is promoted on the (111)B top facet of the underlying NW core (i) while undesired growth on the {1̅1̅0} inclined facets is suppressed (ii). (c) Representative SEM image of the as-grown NW arrays. The image was taken at 45° and tilt-corrected to display actual lengths. (d) TEM micrograph of representative NWs with the intended InGaAs­(Sb) regions highlighted.

The magnified schematic shown in Figure 1b illustrates the underlying growth concept. At the growth front of the GaAs­(Sb) core, three inclined {1̅1̅0} facets, associated with crystal symmetry of the underlying zinc-blende (ZB) structure, emerge from the corners and converge toward the center, forming a triangular plateau on the (111)B top facet.^49,50^ The presence of these inclined facets, together with facet-dependent growth kinetics, governs the subsequent material deposition.^47–51^ The addition of dilute Sb during InGaAs growth is intended to suppress rotational twin formation, thereby enhancing the probability of axial growth on the (111)B top facet of the underlying NW core (i) while minimizing unintended deposition on the {1̅1̅0} inclined facets (ii), which is crucial for achieving a well-defined QD emitter.

Figure 1c presents a representative scanning electron microscopy (SEM) image of the as-grown NW array. Across the investigated field, the NWs exhibited a high yield (≈90%) and uniform morphology with consistent lengths of *L* = (580 ± 40) nm and diameters of *D* = (150 ± 10) nm. Figure 1d shows a TEM micrograph of two NWs mechanically transferred from the same SAE field onto TEM grids, where the intended InGaAs­(Sb) insertion region is indicated by purple triangles.

To assess the axial growth characteristics of the InGaAs­(Sb) insert, high-angle annular dark-field STEM (HAADF–STEM), along with associated EDXS, was performed on multiple representative NWs (see S1. Methods, Supporting Information). Figure 2a presents a HAADF-STEM micrograph of the top region of one NW, where the contrast reflects atomic number (*Z*) variations. The axial AlGaAs/GaAs shell deposition is clearly distinguished with the Al-containing layer appearing darker due to its lower average atomic number. Directly beneath this layer, the InGaAs­(Sb) region (yellow arrow) exhibits brighter contrast relative to the surrounding material due to In incorporation. A high-resolution micrograph in Figure 2b further resolves the region of interest at the NW tip, encompassing the InGaAs­(Sb) insertion. Structural analysis reveals a phase-pure ZB domain free of rotational twins across the entire insert thickness in line with the anticipated surfactant role of dilute Sb in microstructure control.

**Figure 2:**
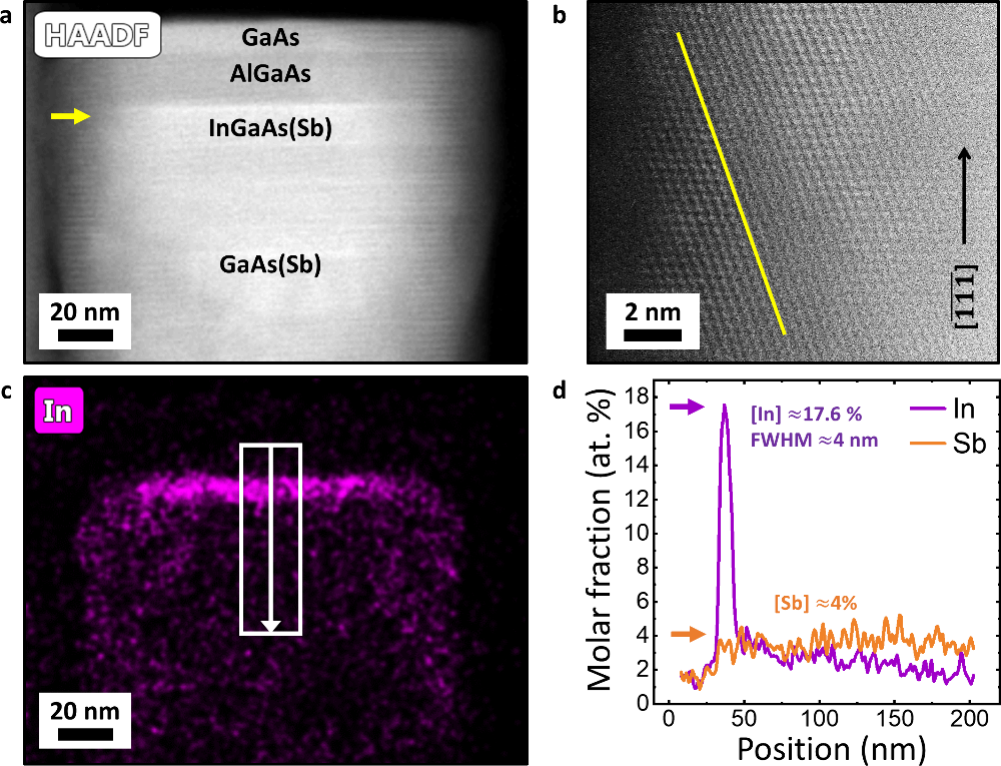
Microstructural and compositional analysis of twin-free InGaAs­(Sb) axial inserts. (a) HAADF-STEM micrograph recorded in the top region of a transferred NW. (b) High-resolution micrograph magnifying the InGaAs­(Sb) region (indicated in (a) by the yellow arrow) with a twin-free ZB domain outlined in yellow. (c) Associated EDXS elemental map of the In distribution, showing an In-rich segment. (d) Vertical compositional profile scanned across the In-rich segment, as indicated by the white arrow in (c). Purple and orange solid lines represent the In and Sb molar fractions, respectively. This plot shows twice the measured atomic fraction of each group III and V element, directly representing the corresponding alloy compositions.

The corresponding EDXS elemental map of In, shown in Figure 2c, reveals a distinct In enrichment localized at the (111)B top facet, forming a thin axially confined region with abrupt heterointerfaces. In contrast, radial In incorporation remains negligible with the measured atomic fraction below the detection limit (less than 1%). This preferential InGaAs­(Sb) deposition at the top facet is consistent with the Sb-mediated suppression of twin formation during growth.

In addition, Figure 2d displays the axial compositional line profile acquired through the center of the In-enriched region, as indicated by the white arrow in Figure 2c. The In profile (purple line) shows that the maximum In content reaches ≈17.6% within the segment with a full width at half-maximum (fwhm) of ≈4 nm, followed by a rapid decay to background levels. This narrow apparent peak width, which is comparable to the effective spatial resolution of the EDXS measurement (≤5 nm), supports the formation of a few-nm-thin insert with abrupt interfaces. The Sb molar fraction (orange line) reaches ≈3–4% within the same segment, as targeted, and remains at a comparable level extending into the GaAs­(Sb) core. These axial growth characteristics of InGaAs­(Sb) are in marked contrast to those typically observed for Sb-free InGaAs segments.^44,45^ As described earlier, Sb-free InGaAs predominantly deposits on the {1̅1̅0} inclined facets rather than on the (111)B top facet of the core, and these segments exhibit a high twin density (≈1.5 nm^–1^), as shown in Figure S2 (Supporting Information).

This distinct behavior can be understood by considering two concurrent and largely independent effects governing the axial growth characteristics of the InGaAs-based segment: (i) stabilized growth on the (111)B top facet, which can be attributed to the Sb surfactant effect,^52,53^ and (ii) growth on the inclined {1̅1̅0} facets, which has mainly been associated with the formation of rotational twins.^47–51^ In the absence of Sb, frequent twin formation together with a less stable axial growth front favors preferential growth on the inclined facets. However, when dilute Sb is introduced, stabilization of the (111)B growth front combined with the reduced twin probability may act synergistically to promote the formation of axially confined InGaAs­(Sb) disks.

Importantly, however, the formation of an axially confined thin disk is not fully deterministic, as twin formation in GaAs-based NWs is inherently stochastic. As shown in Figure S3 (Supporting Information), a subset of NWs within the same array still exhibits variations in the InGaAs­(Sb) deposition profile with partial incorporation occurring on both the top and inclined facets. In these NWs, a twin density of ≈0.5 nm^–1^ is observed, which is about 3-fold lower than in Sb-free InGaAs, although twins are not completely eliminated. Under such conditions, growth on the (111)B top facet remains active, but twin-mediated processes simultaneously lead to additional incorporation on the inclined {1̅1̅0} facets, resulting in an overall slightly larger InGaAs­(Sb) segment volume. While the limited number of NWs analyzed by TEM precludes a statistically rigorous determination of the fraction of twin-free InGaAs­(Sb) inserts across the NW array, the reduced twin density consistently correlates with enhanced axial growth selectivity among the investigated NWs. Further systematic tuning of Sb incorporation and the associated growth conditions may reduce the overall twin density,^53^ thereby enabling the system to approach a statistically deterministic regime for axial QD formation.

To verify the spatial origin and emission properties of the InGaAs­(Sb) insertion, both CL and μPL measurements were performed at low temperature (20 and 4 K, respectively) on transferred single NWs (see S1. Methods in the Supporting Information). It should be noted that these NWs were transferred onto different substrates (SiO_2_/Au/Si and sapphire) from those used for TEM structural analysis (copper grids), and therefore, a direct one-to-one structural-optical correlation cannot be established at this stage. Nevertheless, consistent trends observed across multiple NWs provide reliable insight into their general emission characteristics.

Figure 3a,b displays a CL intensity map of a representative single NW transferred onto a SiO_2_/Au/Si substrate, overlaid on the concurrently acquired secondary-electron (SE) image recorded at 20 K using a 5 kV electron beam, along with the corresponding CL spectrum measured from the same NW. The CL emission is strongly localized at the NW top region, confirming that the luminescence predominantly originates from the InGaAs­(Sb) active segment. Note, in CL, the density of free carriers is substantially higher than in μPL measurements, leading to enhanced spectral diffusion and less pronounced single-emitter features. Accordingly, the detailed spectral features and possible origin of the observed multipeak emission are discussed below based on the higher resolution μPL spectra.

**Figure 3:**
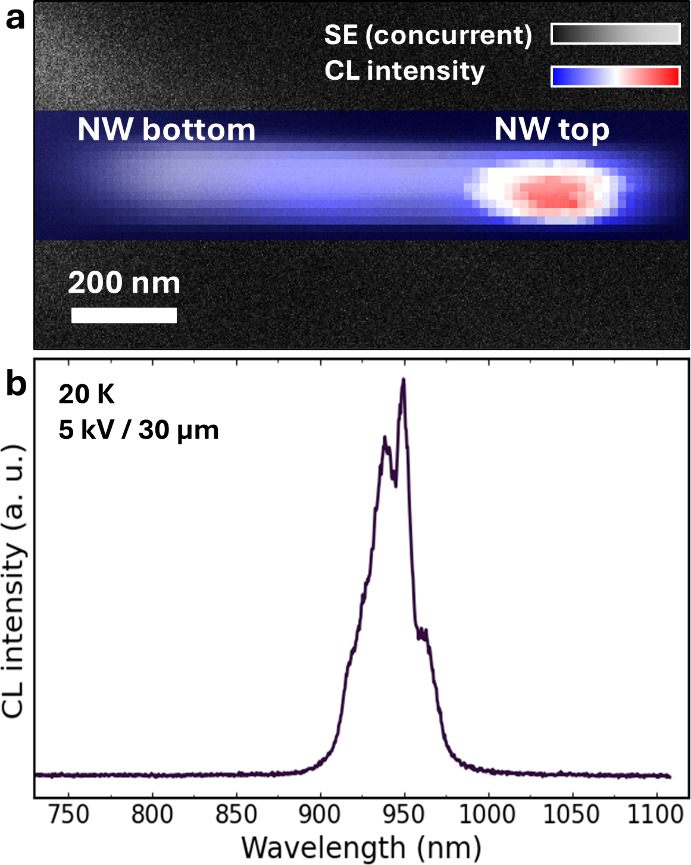
CL measurement of a single InGaAs­(Sb) NW-QD transferred on a SiO_2_/Au/Si substrate. (a) CL intensity map overlaid on the concurrently recorded SE image acquired at 20 K using a 5 kV electron beam and 30 μm beam aperture, showing emission localized at the NW top region corresponding to the InGaAs­(Sb) active segment. (b) The corresponding CL spectrum acquired from the same NW, which exhibits an emission band centered at ≈938 nm.

Figure 4a,b shows excitation power-dependent μPL spectra of representative individual NWs under pulsed excitation. The NWs were transferred onto SiO_2_/Au/Si (a, NW1) and sapphire (b, NW2) substrates, respectively. The SiO_2_/Au/Si substrate primarily serves as a platform for CL measurements, allowing the μPL characteristics to be correlated with the spatial emission origin from the same NWs, while sapphire was also employed as a favorable substrate for high-quality μPL measurements. For NWs on both substrates, emission from the InGaAs­(Sb) insertion was consistently observed. In the representative spectra shown here, the highest-intensity peak is located near ≈1002 nm for NW1 and ≈952 nm for the NW2 sample with a line width (fwhm) of ≈2 nm in both cases. These spectrally well-defined peaks were selected for the corresponding time-resolved PL (TRPL) and second-order photon-correlation measurements discussed below. Although the dominant emission wavelengths differ between the two NWs, both remain within the range expected from the estimated quantum-confined bandgap energy (≈1.25–1.39 eV, corresponding to ≈890–990 nm) obtained from calculations based on the measured dimensions and composition of the InGaAs­(Sb) insertion (see Section S4 of the Supporting Information). Additionally, the characteristic GaAs­(Sb) core emission, expected around 850 nm at low temperature,^44,53^ was completely absent across the investigated NWs, supporting highly efficient transfer to the InGaAs­(Sb) region despite its small volume fraction within the NW (<0.5%).

**Figure 4:**
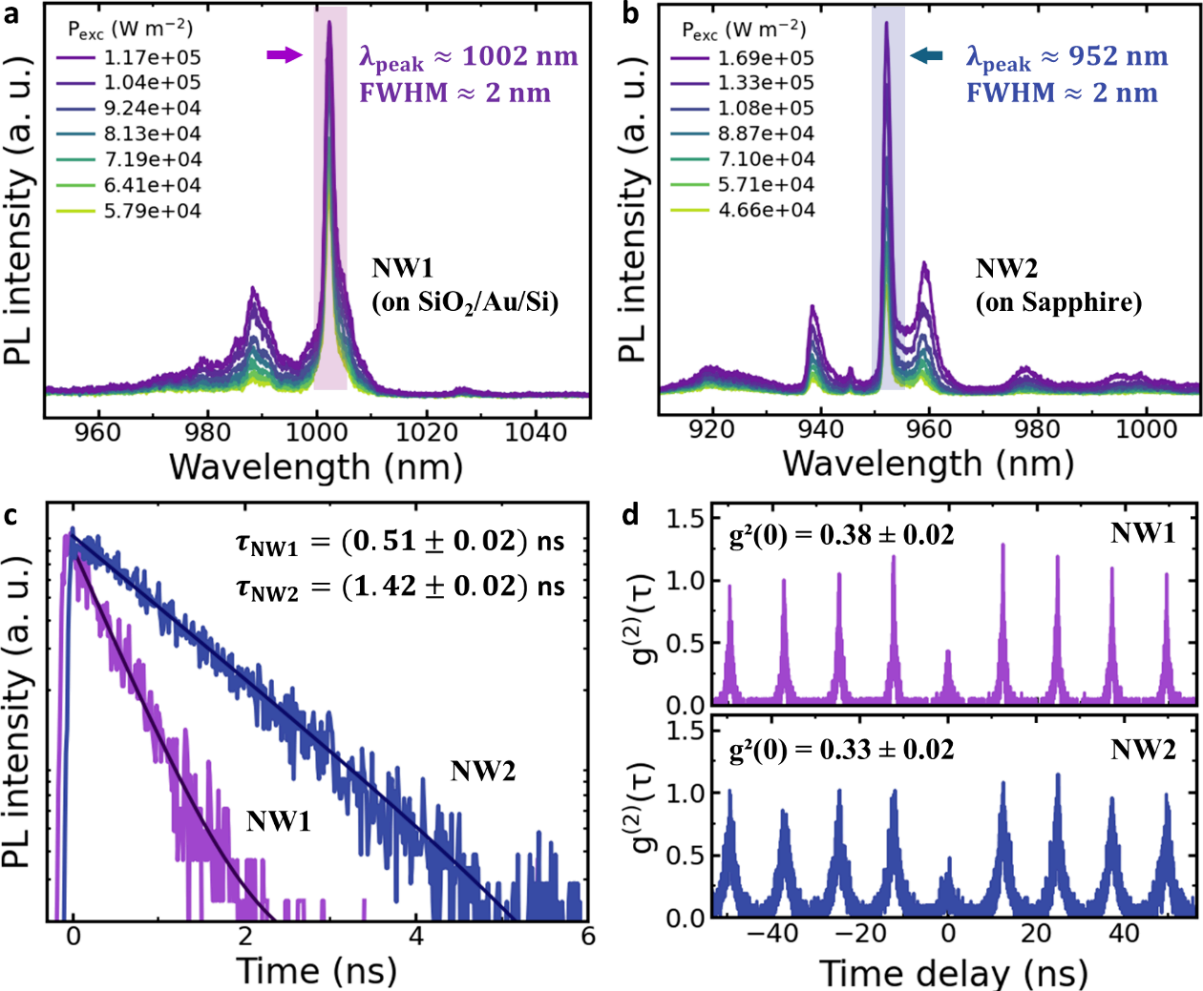
μPL analysis of single InGaAs­(Sb) NW-QDs. (a, b) Excitation power-dependent μPL spectra of single NWs transferred onto SiO_2_/Au/Si (a, NW1) and sapphire (b, NW2) substrates at 4 K, exhibiting emission from the InGaAs­(Sb) insertion. (c) TRPL decay curves measured from NW1 (purple) and NW2 (dark-blue), along with single exponential fits yielding lifetimes of (0.51 ± 0.02) ns and (1.42 ± 0.02) ns, respectively. (d) Pulsed second-order photon-correlation measurements *g* ^(2)^(τ) of the highest-intensity emission peak from NW1 (top) and NW2 (bottom). The pronounced suppression of the zero-delay peak confirms the antibunched photon statistics of single photon emission with *g* ^(2)^(0) of 0.38 ± 0.02 and 0.33 ± 0.02, respectively.

Notably, each InGaAs­(Sb) spectrum shows a multipeak structure with relatively broad emission features rather than a single narrow emission line exhibiting fwhm values below several tens of μeV, as observed in other optimized NW-QD systems.^24–26,28^ This behavior may arise from compositional fluctuations of In and Sb within the InGaAs­(Sb) segment, morphological variations during axial deposition (e.g., growth on both the top and inclined facets), and differences in the local crystal structure (e.g., twin density) as noted above. For example, Section S5 of the Supporting Information provides correlated CL data acquired from the NW1 sample, suggesting that different spectral features can originate from spatially distinct regions within the same NW. More broadly, the majority of the investigated NWs tended to exhibit dominant emission in one of two spectral regimes, centered near ≈1000 nm or ≈950 nm. This distribution may be attributed to a coupled interplay between twin-density-dependent growth facet formation, facet-dependent Sb incorporation, and the influence of Sb incorporation on twin formation. The detailed relationship between these effects and their impact on the optical properties provides a scope for further investigation.

Additionally, Figure 4c shows TRPL decay curves obtained from NW1 (purple) and NW2 (dark-blue), which reveal lifetimes of (0.51 ± 0.02) ns and (1.42 ± 0.02) ns, respectively. In particular, the fast-decaying emitter with ≈1000 nm wavelength emission is representative of lifetimes typical of standard Stranski–Krastanov (SK)-type InGaAs QDs.^5^ The pronounced variation in lifetime between different NW-QDs is consistent with the overall emitter-to-emitter variability observed in the μPL spectra and may reflect differences in local structure and composition, such as In content, segment size or shape, and twin defect density.

To assess the quantum nature of the emission, Figure 4d shows the second-order photon-correlation functions *g* ^(2)^(τ) measured from the selected peak of NW1 (top) and NW2 (bottom), respectively. The measurements were performed under pulsed excitation using a HBT autocorrelation setup integrated with the μPL system (see S1. Methods in the Supporting Information). Photon antibunching behavior is reflected by the suppression of the center peak at zero-time delay in *g* ^(2)^(τ) compared to uncorrelated peaks at τ ≫ 0, indicating sub-Poissonian photon statistics characteristic of nonclassical emission.

The pronounced suppression of the central peak, yielding *g* ^(2)^(0) of 0.38 ± 0.02 for NW1 and 0.33 ± 0.02 for NW2, unambiguously demonstrates the single-photon emission (with well-suppressed multiphoton error) from the individual InGaAs­(Sb) QDs under the small mean photon number assumption. While an ideal single-photon source would exhibit *g* ^(2)^(0) approaching zero, as demonstrated by the near-ideal single-photon purity with *g* ^(2)^(0) < 0.05 reported for optimized NW-QD systems,^24–26^ the relatively large values obtained here can be attributed not only to spectral overlap from adjacent emission lines and residual background emission but also to the unintended structures discussed above and the associated variations in their shape, size, and alloy composition, together with the relatively large lateral dimensions of the NWs. Nevertheless, the *g* ^(2)^(0) values measured from multiple NW samples across different substrates remain well below the conventional threshold of 0.5 for single-photon emitters; for example, even lower values were obtained for selected emitters, including the best raw *g* ^(2)^(0) of 0.27 ± 0.08 and a temporally postselected value of 0.12 ± 0.07, as shown in Section S6 of the Supporting Information. While important challenges remain, including elucidating and controlling the origin of the emission wavelength variability, improving the spectral characteristics, and achieving more deterministic formation of the QD active region, these results nevertheless highlight the strong potential of axial InGaAs­(Sb) NW-QDs as monolithic single photon sources.

In summary, we realized ultrathin axial InGaAs­(Sb) QD-based single photon sources embedded in GaAs-based NWs via catalyst-free SAE growth. By exploiting Sb-mediated suppression of rotational twin formation, axial QD growth on the (111)B top facet is promoted while unintended deposition on the {1̅1̅0} inclined facets is significantly reduced. Structural analysis demonstrates the formation of a few-nm-thin InGaAs­(Sb) region with abrupt compositional transitions and a twin-free ZB domain across the insertion thickness. Single-NW optical spectroscopy reveals intense, spatially localized emission from the InGaAs­(Sb) QD and fast lifetimes as short as (0.51 ± 0.02) ns. Second-order photon-correlation function *g* ^(2)^(τ) measurements under pulsed excitation consistently exhibit pronounced antibunching with raw *g* ^(2)^(0) values below 0.4 and the lowest postselected value of 0.12 ± 0.07, confirming the single-photon nature of the emission despite their still large lateral dimensions and therefore relatively weak lateral confinement. Although the formation of fully twin-free axial inserts remains stochastic, the observed correlation between reduced twin density and preferential axial growth highlights the critical role of surfactant-mediated facet control. Our findings establish Sb-assisted SAE growth as a viable route toward monolithically integrated axial QDs in GaAs-based NWs directly grown on Si-compatible platforms and provide a foundation for further optimization toward statistically deterministic single-photon sources. Future efforts aimed at NW geometry engineering for enhanced carrier confinement, optimized Sb incorporation and associated growth conditions for more deterministic axial QD formation, and integration with on-chip Si photonic waveguides may enable improved optical QD characteristics, controlled emitter alignment, and Purcell-enhanced optical coupling,^17^ advancing scalable NW-based quantum photonic circuits.

## Supplementary Material



## Notes

Methods including NW growth via selective-area molecular beam epitaxy, structural analysis, and optical characterizations; Axial deposition of Sb-free InGaAs; Additional features of axial InGaAs­(Sb) – stochastic nature; Estimation of quantum-confined InGaAs­(Sb) bandgap energy; Additional CL data; Additional second-order correlation measurements (PDF)
