# Supplementary material for: Monolithic Axial InGaAs Quantum Dot Emitters in GaAs-Based Nanowires via Sb-Mediated Facet Engineering
Source: Nano Lett. 2026 Jul 2;26(27):8834–41. doi: 10.1021/acs.nanolett.6c02123 (PMC13377590; doi:10.1021/acs.nanolett.6c02123)
Supplement: Supplementary file 1 [file nl6c02123_si_001.pdf]

Supporting Information for

Monolithic axial InGaAs quantum dot emitters in GaAs-based nanowires via Sb-mediated facet engineering

Hyowon W. Jeong[1,5,], Aris Koulas-Simos[2], Imad Limame[2], Markus Döblinger[3], Sang Kyu Kim[4], Chirag C. Palekar[2], Jonathan J. Finley[1], Stephan Reitzenstein[2], and Gregor Koblmüller[1,2,*]*

[1]Walter Schottky Institute, TUM School of Natural Sciences, Technical University of Munich, Garching 85748, Germany

[2]Institute for Physics and Astronomy, Technical University Berlin, Berlin 10623, Germany

[3]Department of Chemistry, Ludwig-Maximilians-University of Munich, Munich 81377, Germany

[4]Walter Schottky Institute, TUM School of Computation, Information and Technology, Technical University of Munich, Garching 85748, Germany

[5]Department of Physics, University of California, Berkeley, Berkeley, California 94720, United States

*Corresponding authors.

E-mail: hyowon.jeong@berkeley.edu (H.W.J.); gregor.koblmueller@tu-berlin.de (G.K.)

S1. Methods

Nanowire (NW) Growth via Selective-Area Molecular Beam Epitaxy (SAE)

The growth of NWs was performed using a solid-source Gen-II molecular beam epitaxy (MBE) system, equipped with conventional effusion cells for group-III elements (In, Ga, Al) and Veeco valved cracker cells for group-V elements (As, Sb). The As species were supplied as uncracked As_4, and the Sb species as Sb_2 molecules. For the fabrication of SAE nanopatterns, commercial single-side polished 2-inch p-type Si (111) wafers were used as substrates, which were covered by a thermal SiO_2 mask layer (≈20 nm-thick). Patterns of periodic hole opening arrays were written on the SiO_2 mask layer employing electron beam lithography (EBL), reactive ion etching (RIE), and wet chemical etching (buffered hydrofluoric (HF) acid).

Using the prepatterned hole arrays, fully non-catalytic NWs were grown by the growth procedures established in our earlier work.[1-4] First, GaAs(Sb) NWs were grown for 60 min at a substrate temperature of 630°C using Ga flux of 0.35 Å/s, As-BEP (beam equivalent pressure) of 5.5×10^{-5} mbar, and Sb-BEP of 3×10^{-7} mbar. These catalyst-free GaAs(Sb) NWs, which contain only a small Sb molar fraction (≈3–4%), exhibit improved morphological, microstructural, and optical properties due to the so-called Sb-surfactant effect,[3, 4] and therefore serve as stems.

For axially embedding InGaAs-based segments on top of the GaAs(Sb) NW stems, an In-flux of 0.30 Å/s was applied while keeping the Ga- and As-fluxes identical to those used during the NW stem growth, with the substrate temperature ramped down to 590 °C.[5] In this work, the Sb-BEP was intentionally maintained at the same low level (3×10^{-7} mbar) as during the stem growth to suppress twin formation within the active segment, thereby enhancing the likelihood of well-defined axial growth. Finally, $Al_{0.3}Ga_{0.7}As$ (≈5 nm) / GaAs (≈3 nm) passivation layers were coaxially grown at a substrate temperature of 500 °C.[6]

Structural Analysis

The NW growth yield and morphology were evaluated by scanning electron microscopy (SEM) using an NVision 40 FIB-SEM system (Carl Zeiss). Images were acquired at a 45° bird-eye view and tilt-corrected by the SEM software to reflect the actual lengths. High-angle annular dark-field scanning transmission electron microscopy (HAADF-STEM) was performed along the active InGaAs region to characterize the microstructural properties of the NW heterostructures. For these measurements, NWs were mechanically transferred from the SAE array fields onto carbon-coated copper grids and investigated using a FEI Titan Themis TEM operating at 300 kV. Furthermore, energy-dispersive X-ray spectroscopy (EDXS) and corresponding elemental mapping were carried out to analyze the compositional profiles.

Optical characterizations

To identify the spatial origin and emission characteristics of the InGaAs(Sb) axial NW quantum emitters, cathodoluminescence (CL), micro-photoluminescence (μPL), and second-order photon-correlation $g^{(2)}(\tau)$ measurements were performed, as schematically described in **Figure S1**. For CL measurements (a), the NW samples are mounted on a the cold finger of a helium flow cryostat mounted to a high-precision interferometric stage and operated at a temperature of 20 K. Excitation is achieved using a 5 kV electron beam delivered through a 30 μm beam aperture. The emitted photons are collected by a high-numerical aperture parabolic mirror and directed onto the entrance slit (50 μm) of a monochromator. A 300 lines/mm diffraction grating, in combination with a silicon CCD detector, is used to spectrally resolve and record the CL signal on a pixel-by-pixel basis, with an integration time of 50 ms per pixel. Secondary-electron (SE) images were acquired concurrently with the CL measurements to correlate the luminescence with the NW morphology.

The setup for μPL and time-resolved PL (TRPL) measurements (b) consists of a closed-cycle cryostat equipped with a three-axis piezoelectric stage, enabling precise spatial positioning of the sample with nm resolution. Optical excitation is provided by a 80 MHz pulsed laser source via a high-NA (0.62) lens, while the emitted PL is analyzed using a monochromator fitted with a 600 lines/mm grating and detected using a silicon CCD. For TRPL measurements, no deconvolution with the instrumental response function was required, as the decay times are substantially longer than the setup time resolution (≈20 to 30 ps). Photon correlation measurements were performed using a Hanbury Brown and Twiss (HBT) interferometer. The setup incorporates a single-mode fiber beam splitter (BS) to divide the emission into two detection paths, each coupled to a superconducting nanowire single-photon detector (SNSPD). The arrival times of photons are recorded using a time-correlated single-photon counting (TCSPC) module, allowing determination of the second-order autocorrelation function, $g^{(2)}(\tau)$. We extract $g^{(2)}(0)$ by comparing the counts that contribute to the center peak at zero-time delay with the average counts for uncorrelated peaks.

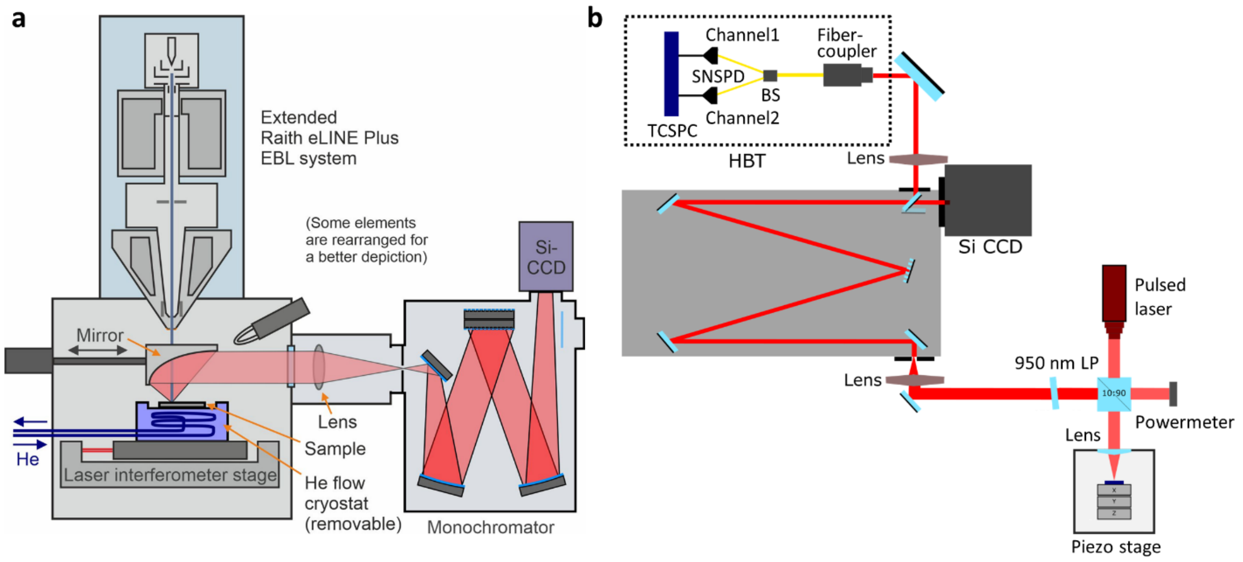

Figure S1: Schematic illustrations of the (a) CL and (b) μPL setups used in this work.

S2. Axial deposition of Sb-free InGaAs

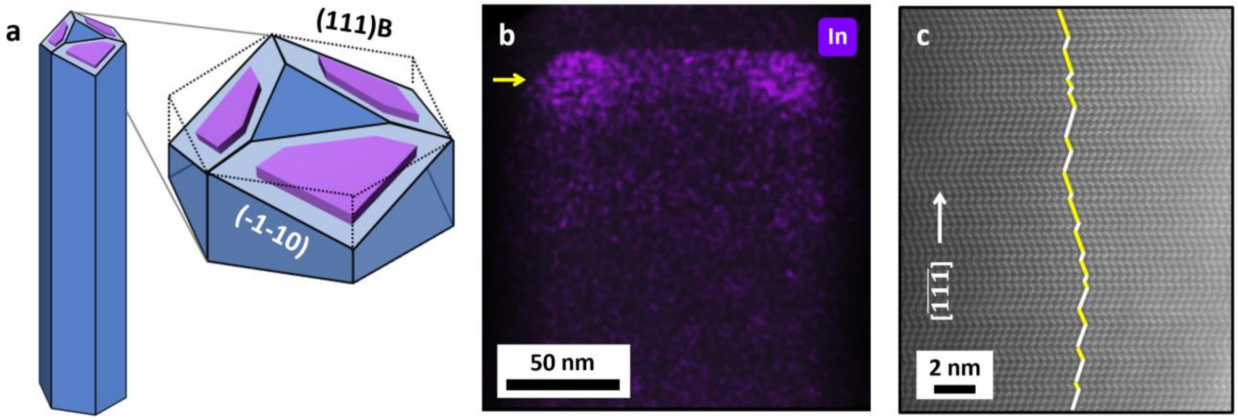

Figure S2: (a) Schematic illustration of axial InGaAs deposition on top of a GaAs(Sb) NW stem, showing that Sb-free InGaAs preferentially grows on the undesired {-1-10} inclined facets rather than on the intended (111)B top facet. (b) Elemental distribution map of In (purple), recorded by EDXS along the InGaAs region, indicating dominant deposition on the inclined facets. (c) High-resolution HAADF-STEM micrograph magnifying the InGaAs region (indicated in (b) by the yellow arrow), where alternating rotational twin domains are outlined in yellow and white (twin density ≈1.5 nm^{-1}). These observations indicate that the frequent formation of twin defects leads to increased growth on the inclined facets. Additionally, the absence of Sb surfactant reduces the stability of the (111)B growth front, further suppressing axial disk formation on the top facet.

S3. Additional features of axial InGaAs(Sb) - stochastic nature

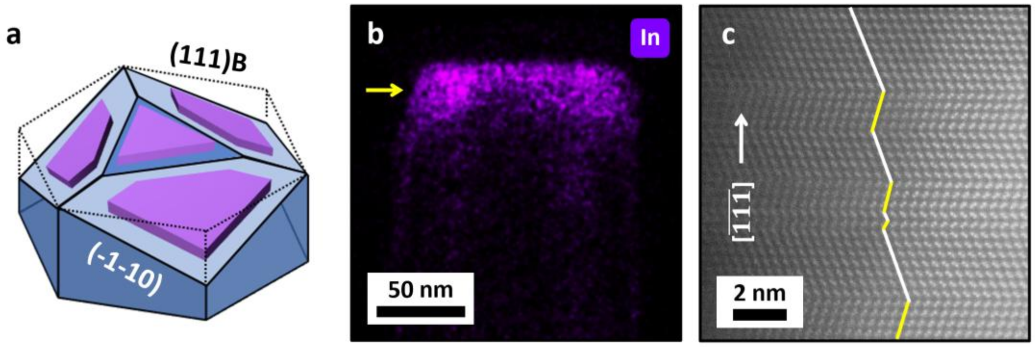

Figure S3: (a) Schematic illustration of axial InGaAs(Sb) deposition on top of a GaAs(Sb) NW stem, showing that, due to its stochastic nature, Sb-containing InGaAs(Sb) grows not only on the intended (111)B top facet but also more dominantly on the undesired {-1-10} inclined facets. (b) Elemental distribution map of In (purple), recorded by EDXS across the InGaAs(Sb) region, indicating deposition on both the top and inclined facets. (c) High-resolution HAADF-STEM micrograph magnifying the InGaAs(Sb) region (indicated in (b) by the yellow arrow), where alternating rotational twin domains are outlined in yellow and white, with a ≈three-fold reduced twin density (≈0.5 nm^{-1}) compared to the Sb-free InGaAs sample (≈1.5 nm^{-1}). These observations indicate that the presence of twins is directly linked to inclined-facet growth. Although Sb surfactant reduces the twin density and stabilizes growth on the (111)B top facet, the stochastic nature of twin formation still leads to a subset of NWs within the array exhibiting unintended growth on the inclined facets. Note that twin formation may not redirect material from the (111)B top facet but rather activates additional incorporation channels on the inclined facets, leading to a larger overall InGaAs(Sb) volume compared to twin-free segments where growth remains confined to the top facet.

S4. Estimation of quantum-confined InGaAs(Sb) bandgap energy

First, assuming a bulk InGaAsSb alloy with [In] = $(20 \pm 2)\%$ and [Sb] = 4%, and using a standard Vegard's law interpolation combined with bowing parameters for the InGaAs[7] and InAsSb sub-systems[8, 9], the estimated bandgap energy at low temperature (0 K) lies in the approximate range

$$E_g^{bulk} \approx 1.18 – 1.23 \text{ eV.}$$

In addition, the bandgap modification induced by quantum confinement can be roughly evaluated using the effective-mass approximation ($m_e^* \approx 0.055\text{m}_0$ and $m_{hh}^* \approx 0.49m_0$ for $In_{0.2}Ga_{0.8}As$ in the ZB structure[10]) and a rectangular quantum box model (infinite potential well). For a simplified rectangular quantum structure with lateral dimensions $L_x = L_y \approx 80$ nm and an active-layer thickness of $L_z \approx 7$ –10 nm (effective confinement width accounting for compositional grading), the electron and heavy-hole confinement energies can be estimated as follows[11].

For the axial direction (here, L_z = 7 nm),

$$E_{e,z} = \frac{\hbar^2\pi^2}{2m_e^* L_z^2} \approx 0.139 \text{ eV,} \qquad E_{hh,z} = \frac{\hbar^2\pi^2}{2m_{hh}^* L_z^2} \approx 0.0156 \text{ eV.}$$

For the lateral direction ($L_x = L_y \approx 80$ nm),

$$E_{e,xy} = \frac{\hbar^2\pi^2}{2m_e^*}\left(\frac{1}{L_x^2} + \frac{1}{L_y^2}\right) \approx 0.00213 \text{ eV,} \qquad E_{hh,xy} = \frac{\hbar^2\pi^2}{2m_{hh}^*}\left(\frac{1}{L_x^2} + \frac{1}{L_y^2}\right) \approx 0.00024 \text{ eV.}$$

The total increase in the interband transition energy due to quantum confinement is therefore

$$\Delta E \approx \left(E_{e,z} + E_{e,xy}\right) + \left(E_{hh,z} + E_{hh,xy}\right) \approx 0.157 \text{ eV.}$$

The resulting quantum-confined bandgap energy (E_g^{Conf}) is estimated to be

$$E_g^{Conf} \approx E_g^{bulk} + \Delta E \approx 1.33–1.39 \text{ eV}$$

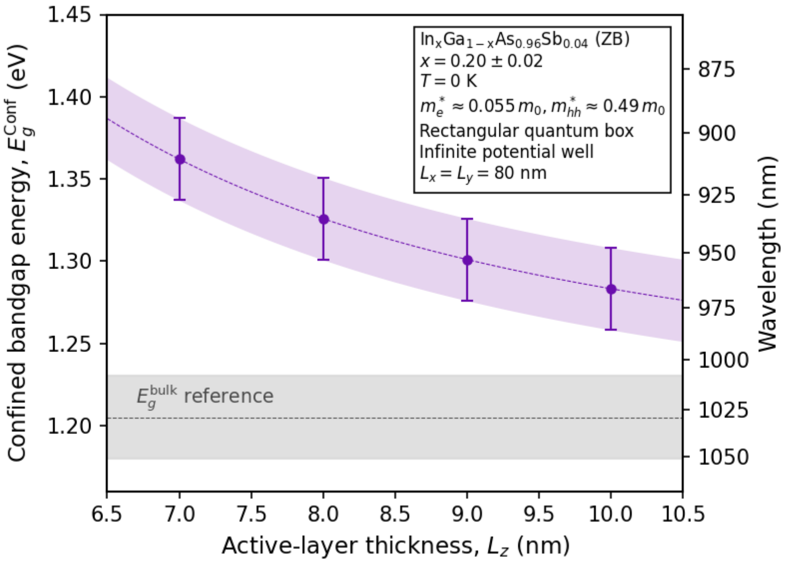

Figure S4: Estimated confined ground state energy (E_g^{Conf}) as a function of active layer thickness (purple), together with the corresponding bulk bandgap energy (E_g^{bulk}, gray) for reference.

Thus, for quantum structures with a thickness of $L_z \approx 7$–10 nm and lateral dimensions of $L_x = L_y \approx 80$ nm, the confined ground state energy is estimated to lie in the range

$$E_g^{Conf} \approx 1.25\text{–}1.39 \text{ eV},$$

corresponding to wavelengths of $\lambda \approx 890$–990 nm, as summarized in **Figure S4**.

Although additional bandgap shifts arising from strain within the NW, variations in twin defect density, and local compositional (both In and Sb) or structural fluctuations should ideally be taken into account, this order-of-magnitude estimate nevertheless yields transition energies comparable to the typical range of experimentally observed PL peak energies. It should also be noted that this model assumes an infinite potential well and fixed effective masses, therefore, the calculated value likely represents an approximate upper limit on the confinement-induced blue shift relative to the bulk bandgap.

S5. Additional CL data

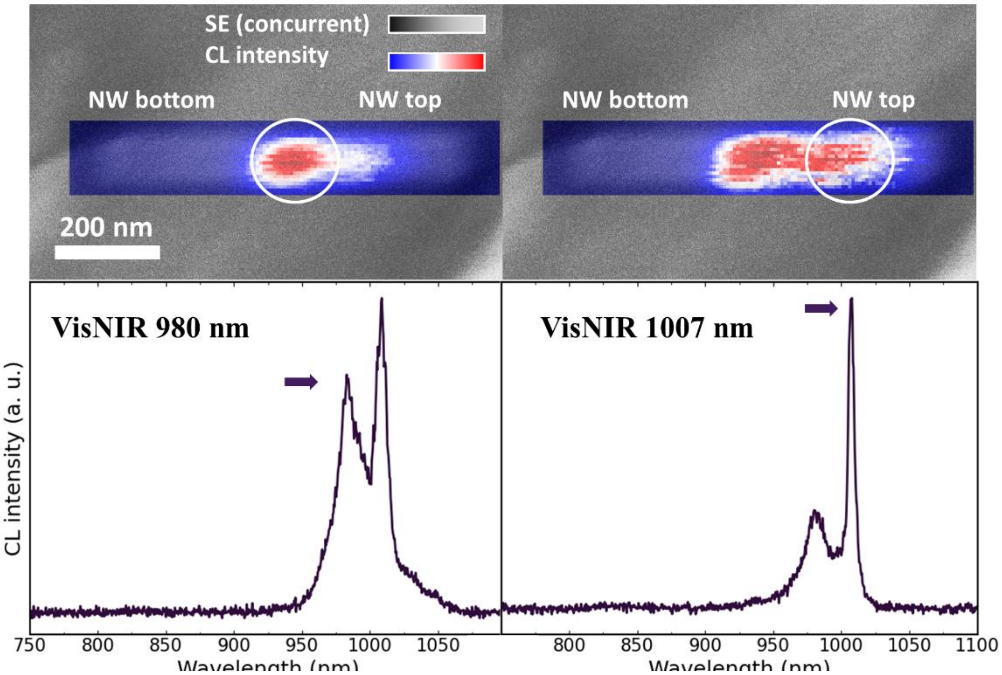

Figure S5: CL intensity maps recorded using spectral windows centered at 980 nm (left) and 1007 nm (right), along with the corresponding CL spectra extracted from the same NW. The emission detected around 980 nm is mainly localized near the central region of the NW, whereas the 1007 nm emission, corresponding to the spectral range where pronounced antibunching with $g^{(2)}(0)$ of 0.38±0.02 was observed in μPL, is concentrated closer to the NW tip region. These data provide supportive evidence that the single-photon emission is associated with the InGaAs(Sb) insertion near the NW tip, although the two spectral contributions cannot be fully spatially isolated in the CL maps due to the spectral overlap of these emitters.

S6. Additional second-order correlation measurements

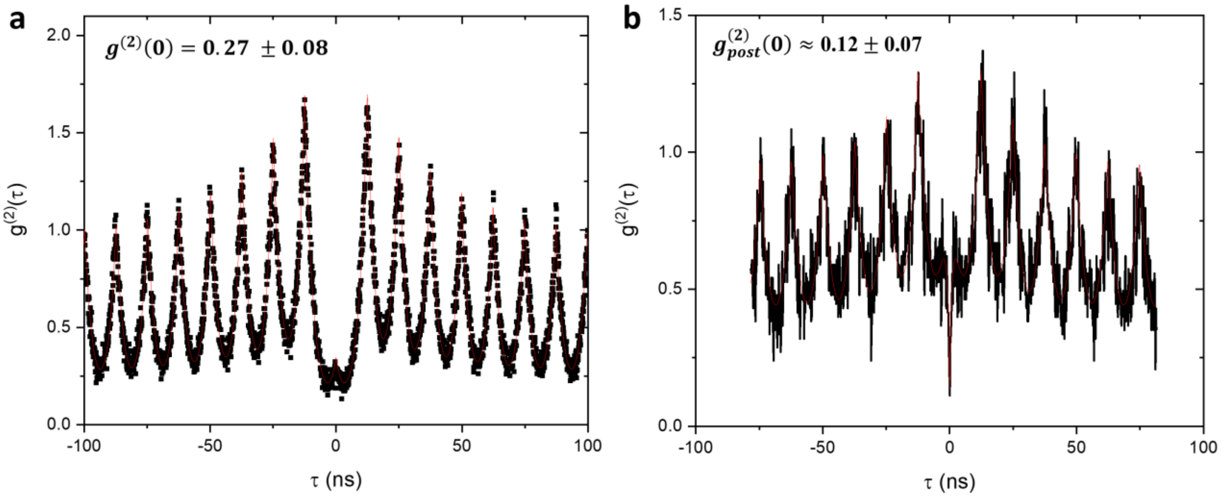

Figure S6: (a) Second-order photon-correlation function $g^{(2)}(\tau)$ measured under pulsed excitation, yielding a raw $g^{(2)}(0)$ value of 0.27±0.08. The suppressed zero-delay peak confirms antibunching, while the weak bunching envelope ($g^{(2)}(\tau) > 1$) extending over multiple pulse periods suggests the presence of additional slow emitter state dynamics, such as charge trapping, recapture, or blinking-related effects. (b) $g^{(2)}(\tau)$ data from another emitter, yielding $g^{(2)}(0)$ = 0.12±0.07 after temporal post-selection. In this case, the long radiative lifetime leads to substantial overlap between neighboring peaks, causing a pronounced background around zero delay. Therefore, the post-selected value corresponds to the minimum at zero delay, such that only photons emitted immediately after the excitation pulse are counted, while delayed emission associated with recaptured carriers is excluded.

REFERENCES

[1] Ruhstorfer, D.; Mejia, S.; Ramsteiner, M.; Döblinger, M.; Riedl, H.; Finley, J. J.; Koblmüller, G. Demonstration of n-type behavior in catalyst-free Si-doped GaAs nanowires grown by molecular beam epitaxy. *Appl. Phys. Lett.* **2020**, *116* (5), 052101.

[2] Ruhstorfer, D.; Lang, A.; Matich, S.; Döblinger, M.; Riedl, H.; Finley, J. J.; Koblmüller, G. Growth dynamics and compositional structure in periodic InAsSb nanowire arrays on Si (111) grown by selective area molecular beam epitaxy. *Nanotechnology* **2021**, *32* (13), 135604.

[3] Ajay, A.; Jeong, H.; Schreitmüller, T.; Döblinger, M.; Ruhstorfer, D.; Mukhundhan, N.; Koolen, P. A. L. M.; Finley, J. J.; Koblmüller, G. Enhanced growth and properties of non-catalytic GaAs nanowires via Sb surfactant effects. *Appl. Phys. Lett.* **2022**, *121* (7), 072107.

[4] Jeong, H. W.; Ajay, A.; Yu, H.; Döblinger, M.; Mukhundhan, N.; Finley, J. J.; Koblmüller, G. Sb-mediated tuning of growth- and exciton dynamics in entirely catalyst-free GaAsSb nanowires. *Small* **2023**, *19* (16), 2207531.

[5] Jeong, H. W.; Ajay, A.; Döblinger, M.; Sturm, S.; Gómez Ruiz, M.; Zell, R.; Mukhundhan, N.; Stelzner, D.; Lähnemann, J.; Müller-Caspary, K.; Finley, J. J.; Koblmüller, G. Axial growth characteristics of optically active InGaAs nanowire heterostructures for integrated nanophotonic devices. *ACS Appl. Nano Mater.* **2024**, *7* (3), 3032–3041.

[6] Rudolph, D.; Funk, S.; Döblinger, M.; Morkötter, S.; Hertenberger, S.; Schweickert, L.; Becker, J.; Matich, S.; Bichler, M.; Spirkoska, D.; Zardo, I.; Finley, J. J.; Abstreiter, G.; Koblmüller, G. Spontaneous alloy composition ordering in GaAs-AlGaAs core-shell nanowires. *Nano Lett.* **2013**, *13* (4), 1522–1527.

[7] Khomyakov, P. A.; Luisier, M.; Schenk, A. Compositional bowing of band energies and their deformation potentials in strained InGaAs ternary alloys: A first-principles study. *Appl. Phys. Lett.* **2015**, 107, 062104.

[8] Murawski, K.; Gomółka, E.; Kopytko, M.; Grodecki, K.; Michalczewski, K.; Kubiszyn, Ł.; Gawron, W.; Martyniuk, P.; Rogalski, A.; Piotrowski, J. Bandgap energy determination of InAsSb epilayers grown by molecular beam epitaxy on GaAs substrates. *Prog. Nat. Sci.: Mater. Int.* **2019**, 29, 472–476.

[9] Svensson, S. P.; Sarney, W. L.; Hier, H.; Lin, Y.; Wang, D.; Donetsky, D.; Shterengas, L.; Kipshidze, G.; Belenky, G. Band gap of $InAs_{1-x}Sb_x$ with native lattice constant. *Phys. Rev. B* **2012**, 86, 245205.

[10] Goldberg, Y. A.; Shmidt, N. M. Gallium Indium Arsenide ($Ga_xIn_{1-x}As$). *In Handbook Series on Semiconductor Parameters*, Vol. 2; Levinshtein, M.; Rumyantsev, S.; Shur, M., Eds.; World Scientific: London, **1999**, 62–88.

[11] Harrison, P.; Valavanis, A. *Quantum Wells, Wires and Dots: Theoretical and computational physics of semiconductor nanostructures.* Wiley, **2016**, 254–255
